# *PTEN* Sequence Analysis in Endometrial Hyperplasia and Endometrial Carcinoma in Slovak Women

**DOI:** 10.1155/2015/746856

**Published:** 2015-05-31

**Authors:** H. Gbelcová, P. Bakeš, P. Priščáková, V. Šišovský, I. Hojsíková, Ľ. Straka, M. Konečný, J. Markus, C. W. D'Acunto, T. Ruml, D. Böhmer, Ľ. Danihel, V. Repiská

**Affiliations:** ^1^Institute of Medical Biology, Genetics and Clinical Genetics, Faculty of Medicine and University Hospital Bratislava, Comenius University in Bratislava, Sasinkova 4, 81108 Bratislava, Slovakia; ^2^Department of Biochemistry and Microbiology, Faculty of Food and Biochemical Technology, University of Chemistry and Technology, Prague, Technicka 5, 16628 Prague, Czech Republic; ^3^Institute of Pathological Anatomy, Faculty of Medicine and University Hospital Bratislava, Comenius University in Bratislava, Bratislava, Sasinkova 4, 81108 Bratislava, Slovakia; ^4^MEDIREX GROUP ACADEMY n.o., Galvaniho 17/C, 82016 Bratislava, Slovakia; ^5^Clinical Pathology Presov, Ltd., Holleho 14, 08001 Presov, Slovakia; ^6^Department of Clinical Genetics, St. Elizabeth Cancer Institute, Heydukova 10, 812 50 Bratislava, Slovakia

## Abstract

Phosphatase and tensin homolog (PTEN) is a protein that acts as a tumor suppressor by dephosphorylating the lipid second messenger phosphatidylinositol 3,4,5-trisphosphate. Loss of PTEN function has been implicated in the pathogenesis of a number of different tumors, particularly endometrial carcinoma (ECa). ECa is the most common neoplasia of the female genital tract. Our study evaluates an association between the morphological appearance of endometrial hyperplasia and endometrial carcinoma and the degree of *PTEN* alterations. A total of 45 endometrial biopsies from Slovak women were included in present study. Formalin-fixed and paraffin-embedded tissue samples with simple hyperplasia (3), complex hyperplasia (5), atypical complex hyperplasia (7), endometrioid carcinomas G1 (20) and G3 (5), and serous carcinoma (5) were evaluated for the presence of mutations in coding regions of *PTEN* gene, the most frequently mutated tumor suppressor gene in endometrial carcinoma. 75% of the detected mutations were clustered in exons 5 and 8. Out of the 39 mutations detected in 24 cases, 20 were frameshifts and 19 were nonsense, missense, or silent mutations. Some specimens harboured more than one mutation. The results of current study on Slovak women were compared to a previous study performed on Polish population. The two sets of results were similar.

## 1. Introduction

*Endometrial cancer *is the most common type of uterine cancer and occupies the fourth place among all cancers among developed countries [1, 2]. Noncancerous changes of endometrium are commonly known as hyperplasia.* Endometrial hyperplasia* essentially implies overgrowth of endometrium. It is almost exclusively associated with a relative excess of endogenous or exogenous estrogen. Simple hyperplasia (SH) resembles the normal endometrial tissue growth pattern, while* complex hyperplasia* (*CH*) has a more complex and thus more abnormal architectural growth pattern. Both simple and* complex hyperplasia* can be associated* with cellular atypia* (SAH,* CAH*), which seems to be the most important predictor of malignant potential. There are two basic types of endometrial carcinoma (ECa):* endometrioid* (estrogen related, indolent behaviour) and* nonendometrioid* (unrelated to estrogen, aggressive). Endometrial cancer cells are described as well differentiated, Grade 1 (ECG1), moderately differentiated, Grade 2 (ECG2), or poorly differentiated, Grade 3 (ECG3).* Serous carcinoma* (SC) represents an example of nonendometrioid carcinoma and it is automatically classified as Grade 3 due to its high aggressiveness [3].

After some years of study, endometrial carcinoma still shows the highest percentage of* PTEN* (the phosphatase and tensin homolog, also called* MMAC1* and* TEP1*, MIM 601728) mutations of all tumor types [4]. The tumor suppressor gene* PTEN* was identified on chromosome 10p23.31. It encodes a 403-amino acid PTEN protein (47 kDa) with the activity of phosphatase that can act on both polypeptide and phosphoinositide substrates [5–7]. The structure of PTEN consists of an N-terminal phosphatase domain and a C2 domain: the phosphatase domain contains the active site, responsible for the enzymatic function of the protein, while the C2 domain binds the membrane phospholipids [7]. Thus PTEN binds the membrane through its C2 domain, bringing the active site to the membrane-bound phosphatidylinositol 3,4,5-triphosphate (PIP3) in order to dephosphorylate it. PIP3, the PTEN primary target, is involved in a signal transduction pathway that regulates cell growth, migration, and apoptosis [8, 9]. Loss of heterozygosity at the 10q23.3 locus, PTEN somatic mutations, and changes in the levels and distribution of proteins in the PTEN-PI3K/Akt signal transduction pathway were shown to associate with endometriosis [10].

*PTEN* mutation rate in ECa and hyperplasia is well documented in many populations, but not in the Slovak one. The purpose of this study was the assessment of the quality and frequency of* PTEN* gene mutations in endometrial hyperplasia and endometrial carcinoma in biopsies taken from Slovak women and to compare the results with polymorphism of another Slavic (Polish) and worldwide populations.

## 2. Materials and Methods

### 2.1. Collection and Histopathological Identification of Specimens

A total of 45 archived (from the Department of Pathology, Faculty of Medicine, Comenius University in Bratislava, University Hospital Bratislava (during the years 1997–2011), from the Klinicka patologia Presov, s.r.o. and from the Cytopathos, s.r.o.) formalin-fixed and paraffin-embedded human biopsy hysterectomy and curettage tissue specimens (from uterus of Slovak women hospitalized at Clinics of Gynecology and Obstetrics, Faculty of Medicine, Comenius University in Bratislava, University Hospital Bratislava) were classified by light microscope [11] as SH (3x), CH (5x), ACH (7x), ECG1 (20x), ECG3 (5x) and SC (5x) histological subset of endometrial carcinoma, and serous (SC, 5x) (prototypic endometrial carcinoma type II) histological subset of endometrial carcinoma. ECG2 samples were not included due to nonhomogeneous histological constitutions; they mostly are overlaid with the grade of histological differentiation G3. The procedures of the study received ethics approval from the Ethics Committee of Faculty of Medicine, Comenius University in Bratislava, Slovakia, responsible for the human experimentation. Date of approval is 9 July 2007.

### 2.2. Detection of* PTEN* Gene Mutations

Genomic DNA from all 45 cases was isolated from microdissected cryostat sections of biopsy tissue specimens by QIAamp Micro Kit (Qiagen Manchester Ltd., Manchester, UK). All nine exons of* PTEN* were amplified separately. The sequences of primers for the amplification of exons are

 exon 1 fwd.: 5′-CAGAAGAAGCCCCGCCACCAG-3′,
 exon 1 rev.: 5′-AGAGGAGCAGCCGCAGAAATG-3′, (177-bp amplicon);
 exon 2 fwd.: 5′-TTTCAGATATTTCTTTCCTTA-3′,
 exon 2 rev.: 5′-AACATGAATATAAACATCAA-3′, (171-bp amplicon);
 exon 3 fwd.: 5′-TAATTTCAAATGTTAGCTCAT-3′,
 exon 3 rev.: 5′-AAGATATTTGCAAGCATACAA-3′, (147-bp amplicon);
 exon 4 fwd.: 5′-GTTTGTTAGTATTAGTACTTT-3′,
 exon 4 rev.: 5′-ACAACATAGTACAGTACATTC-3′, (150-bp amplicon);
 exon 5 fwd.: 5′-TATTCTGAGGTTATCTTTTTA-3′,
 exon 5 rev.: 5′-AGGAAAAACATCAAAAAATAA-3′, (292-bp amplicon);
 exon 6 fwd.: 5′-TTGGCTTCTCTTTTTTTTCTG-3′,
 exon 6 rev.: 5′-ACATGGAAGGATGAGAATTTC-3′, (202-bp amplicon);
 exon 7 fwd.: 5′-CCTGTGAAATAATACTGGTATG-3′,
 exon 7 rev.: 5′-CTCCCAATGAAAGTAAAGTACA-3′, (229-bp amplicon);
 exon 8 fwd.: 5′-TTAAATATGTCATTTCATTTCTTTTTC-3′,
 exon 8 rev.: 5′-ACACATCACATACATACAAGTC-3′, (331-bp amplicon);
 exon 9 fwd.: 5′-TTCATTTTAAATTTTCTTTCT-3′,
 exon 9 rev.: 5′-TGGTGTTTTATCCCTCTTGAT-3′, (242-bp amplicon).

PCR amplifications were performed in 50-*μ*L reaction volumes containing 150–200 ng of genomic DNA, 25 mM MgCl_2_ (Roche, Germany), 10 mM each of dGTP, dATP, dTTP, and dCTP, 0.5 *μ*M of each primer (Sigma-Genosys, Lambda Life, Slovakia), and 5 units of FastStart Taq DNA Polymerase (Roche, Germany), 2.5 *μ*L of buffer without Mg^2+^ for Taq DNA polymerase (Roche, Germany), and nuclease-free water to a total volume of 50 *μ*L.

After the denaturing step at 95°C for 10 minutes, 40 cycles of denaturation at 94°C for 15 s, annealing for 20 s, and elongation at 72°C for 30 s were performed, followed by final elongation at 72°C for 10 minutes. Annealing temperatures were as follows: 65.1°C for exon 1, 45.3°C for exon 2, 50.8°C for exons 3, 5, 7, and 9, 44.9°C for exon 4, 56.1°C for exon 6, and 53.4°C for exon 8.

PCR fragments were purified by ExoSAP-It PCR Product Clean Up (Affymetrix, California, USA) as described by the manufacturer and prepared for automated sequencing analysis using BigDye Terminator v. 1.1 Cycle Sequencing Kit (Applied Biosystems, California, USA). Before sequencing by using ABI PRISM 310 Genetic Analyzer (Applied Biosystems, California, USA) samples were purified by ExTerminator kit (Ecoli, Bratislava, Slovakia) as described by the manufacturer. The individual sequences were compared against the reported genomic sequence of* PTEN* using Chromas ver. 2.33. (Technelysium Pty Ltd.).

### 2.3. Statistical Analysis

The statistical analysis of the results was carried out using Fisher's exact test and was performed using IBM SPSS Statistics software ver. 20. *P* values <0.05 were considered statistically significant.

## 3. Results

We screened all nine exons of the* PTEN* gene in 15 hyperplasias and 30 endometrial cancers. We found 39 mutations in 24 of these specimens. We detected mutations in 3 (60%) of 5 complex hyperplasias and in 2 (29%) of complex hyperplasias with cellular atypia. None of 3 simple hyperplasia samples contained mutations. In the series of 25 endometrioid carcinomas, we detected mutations in 15 (75%) of 20 ECG1 and in 4 (80%) of 5 ECG3. However, none of 5 serous adenocarcinomas contained mutations. These data are presented in Table 1.

The qualities of changes found in* PTEN* gene were highly diverse. Out of the 39 mutations, 20 were frameshifts, and the remaining 19 were single base substitutions. All 20 frameshift mutations were predicted to create new stop codons and produce truncated protein products. Over the 19 single base substitution mutations, 2 were nonsense mutations resulting in new stop codons, 12 were missense mutations resulting in single amino acid substitution, and 5 were silent mutations resulting in no amino acid change. Of the 15 samples of ECG1 that contained mutation, 6 (40%) cases harbored more than one mutation (one specimen harbored 2 mutations in one exon). Of the 4 samples of ECG3 that contained mutation, 1 (25%) case harbored more than one mutation. All identified changes in* PTEN* gene are detailed in Table 2.

**Figure 1:**
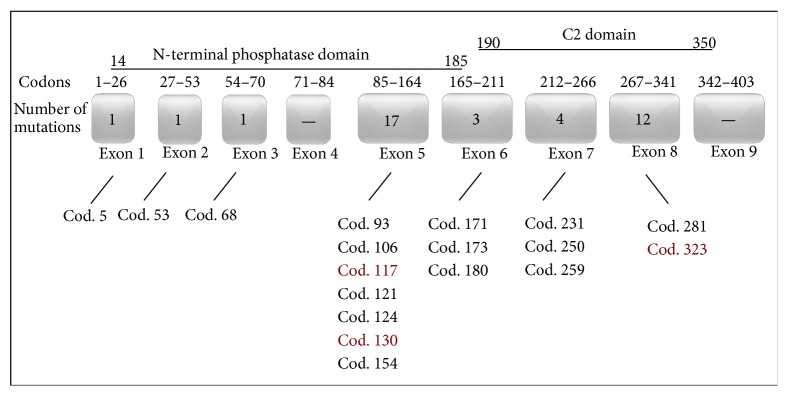
Spectrum of* PTEN* mutations in Slovak women.

Detected mutations were distributed in the following manner: 17 mutations in exon 5; 12 mutations in exon 8; 4 mutations in exon 7; 3 mutations in exon 6; and 1 mutation in each of exons 1, 2, and 3. No mutations were detected in exons 4 and 9. Nonspecified heterozygous deletion was disclosed in the place of annealing reverse primer for exon 9 in most of tested samples. Spectrum of* PTEN* mutations is shown in Figure 1.

Mutations in* PTEN* gene were most frequent in exon 5 (17/44%), mainly in codon 130 which had abnormalities in seven samples. All cases were missense mutations; four of them were insertions of one deoxyadenosine in codon 117 creating stop codon. Both these mostly occurred mutations in exon 5 localized in region encoding the N-terminal domain of PTEN protein.

**Figure 2:**
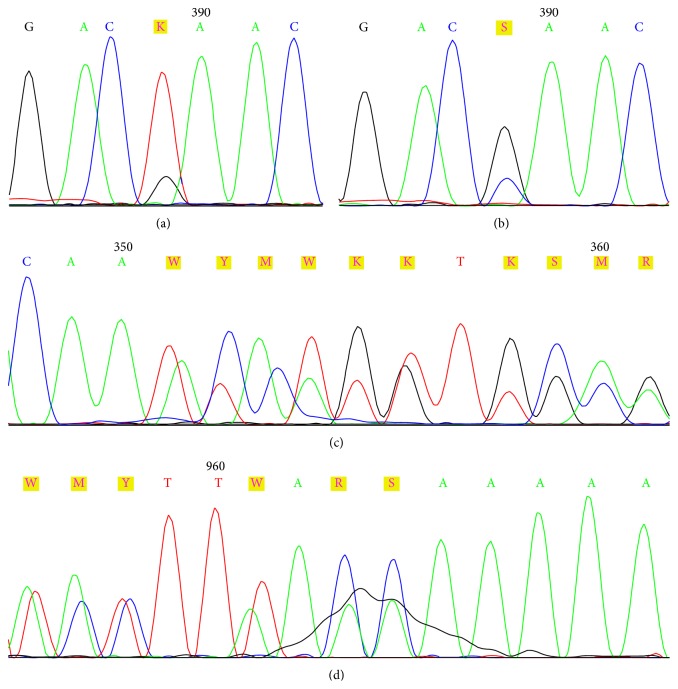
DNA sequencing histograms from three patients with the most frequent mutations of the* PTEN* gene. (a) Substitution in codon 130, G > T, forward primer used; (b) Substitution in codon 130, G > C, forward primer used; (c) A insertion in codon 117, forward primer used; (d) A deletion in codon 323, reverse primer used.

Another site showing a higher (12/38%) frequency of mutations was exon 8. In eleven samples, an identical deletion of deoxyadenosine was present in codon 323, resulting in the formation of stop codon in position 343. This mutation occurred in the region encoding the C2 domain. To conclude, of the 39 mutations disclosed in* PTEN* gene, 29 (75%) clustered in exons 5 and 8. The frequency of the mutations is reported in Table 3. The examples of DNA sequencing histograms with the most frequent mutations are shown in Figure 2.

## 4. Discussion

*PTEN* gene mutations in endometrial carcinomas and hyperplasia were numerous and varied widely in their quality. In 24 out of 45 endometrial samples (53.3%), 39 mutations in* PTEN* gene were identified and most of them (22/39; 56.4%) led to the synthesis termination of encoded protein. The most frequently observed mutations were in exons 5 and 8. One deletion hot spot of one adenosine residue in codon 323 of exon 8 has been identified. The other two hot spot mutations were in exon 5, namely, missense substitution in codon 130 and insertion of one nucleotide in codon 117.

The current study is, according to the authors' knowledge, the only one comparing the* PTEN* gene polymorphism in hyperplasia and endometrial carcinomas in the population of Slovak women. Statistical evaluation of the data obtained has certain limitations due to the small population of Slovakia and very small number of patients in some groups. Consequently, it is difficult to interpret the results from such a small number. However, our study includes all available and useful samples from Slovak women.

The prevalence of mutations in hot spots was similar between women from Slovakia and United States [12–18]. In contrast with the missense mutation in codon 130 (exon 5) that was frequently found also in endometrial cancer of Polish women (12/35; 34%), hot spot for mutation 323 in exon 8 showed different pattern between the Slovak and Polish populations. Only one insertion and one deletion (2/35; 5.7%) at the 6-bp repeat of adenine nucleotides in codons 321–323 have been identified in Polish population [19]. Moreover, data from Japan revealed rare number of mutations in codon 323 in exon 8 and no point mutation in codon 130 [20, 21]. Interestingly, transitions in codon 233, resulting in a nonsense mutation, were detected in 8 cases in Japan (8/25; 32%) [20, 21], in 3 cases in United States (3/134; 2.2%) [12–18], and in 3 cases in Poland (3/35; 8.5%) [19], but the present study revealed no point mutations in codon 233 in Slovak women. Finally, the insertion of one nucleotide in codon 117 frequently occurring in Slovak women samples was not at statistically significant value in any other population studied.

It was previously reported that mutations occurring in* PTEN* exons encoding the phosphatase domain cause complete loss of its suppressor function thus leading more rapidly to the development of a more malignant phenotype of endometrial tumors [22]. In our study 59% (23/39) of mutations described were localized in N-terminal phosphatase domain. The rest of detected mutations (16/39; 41%) were localized in* PTEN* gene region encoding C2 domain. As suggested by Konopka et al. the mutations in* PTEN* gene region encoding C2 domain may develop in the initial stages of neoplastic process but participation of other genes is indispensable to accelerate its progression [22]. No mutation has been discovered beyond the regions encoding N-terminal phosphatase and C2 domains. Similar trend in distribution of mutations in* PTEN* gene was described also in Polish population (65% in N-terminal phosphatase domain, 31.4% in C2 domain) [19, 22], in contrast with the opposite distribution of* PTEN* gene mutations observed in the study from United States (47% in N-terminal phosphatase domain, 50% in C2 domain) [12–18] and Japan (33% in N-terminal phosphatase domain, 66% in C2 domain) [20, 21].

*PTEN* mutations have been shown to occur in about 25% of endometrial hyperplasias and in up to 80% of endometrioid endometrial carcinomas [4]. In the present study, provided on biopsies from Slovak women, we found a similar tendency.* PTEN* mutations were disclosed in 33% of hyperplasias and in 76% of endometrioid carcinomas. Konopka and coworkers demonstrated the presence of mutations in the* PTEN* gene in 45.8% of endometrial carcinomas in Poland [23]. In Japanese population, the prevalence of* PTEN* mutations detected in endometrioid carcinomas was 22.8% [21]. However, in all the studied populations, the prevalence of* PTEN* mutations in hyperplasias was even lower compared to endometrioid carcinoma, namely, 33.3% in Poland [23] and 9.6% in Japan [21]. As summarized in Table 4 there are significant differences in frequency of* PTEN* gene mutations in Slovak and Japanese women. On the other hand, the results received in our study show statistically significant similarity of* PTEN* mutations distribution and their link to endometrial cancer in Slovak and Polish women. It remains a question whether this is due to genetic similarity or there is a territorial or lifestyle link among these groups. In 2009 there was 15.7% of obese women in Polish population, 15.2% in Slovak population, and 3.5% in Japanese population [24]. Significant differences, with similar trend, were also in the percentage of the overweight women (Poland, 29.4%; Slovakia, 27.3%; and Japan, 17.3%) [24]. In the same year, the consumption of meat/fish was as follows (kg/capita/year): Poland 75/12, Slovakia 60/8, and Japan 46/54 [25]. The different diets in the Slovak, Polish, and Japanese nations, with special regard to the consumption of meat versus fish and average body weight parameters between the mentioned populations, correlate well with the trend in the incidence of EC among these nations. However it has been recently published that there are no findings to support an association between meat and fish intakes [26].

Studies on genetic defects participating in endometrial carcinogenesis mechanisms may be useful for prognostic and predictive purposes. In the future, novel therapies may be based on findings from these types of studies and therefore those studies may have a long term clinical importance.

## Figures and Tables

**Table 1:** Frequency of *PTEN* mutations in endometrial samples.

Histologic diagnosis	Mutation frequency (%)
Hyperplasias	5/15 (33%)
Simple hyperplasia	0/3 (0%)
Complex hyperplasia	3/5 (60%)
Atypical complex hyperplasia	2/7 (29%)
Endometrioid carcinomas	19/25 (76%)
Grade 1	15/20 (75%)
Grade 3	4/5 (80%)
Nonendometrioid carcinomas	0/5 (0%)
Serous adenocarcinoma	0/5 (0%)

**Table 2:** List of *PTEN* mutations in endometrial samples.

Case number	Exon	Nucleotide	Codon	Base change	Predicted effect	Histological diagnosis
1	6	540	180	Del G	Stop 181	Complex hyperplasia

2	7	751	250	TGT to CGT	Cys to Arg	Complex hyperplasia

3	8	968	323	Del A	Stop 343	Complex hyperplasia

4	5	349–351	117	Ins A	Stop 125	Atypical complex hyperplasia
	7	691–693	231	Del C	Stop 255	
	8	968	323	Del A	Stop 343	

5	5	389	130	CGA to CTA	Arg to Leu	Atypical complex hyperplasia
	8	968	323	Del A	Stop 343	

6	8	968	323	Del A	Stop 343	Endometrioid carcinoma Grade 1

7	5	388	130	CGA to GGA	Arg to Gly	Endometrioid carcinoma Grade 1

8	5	278	93	Del A	Stop 98	Endometrioid carcinoma Grade 1

9	8	968	323	Del A	Stop 343	Endometrioid carcinoma Grade 1

10	5	349–351	117	Ins A	Stop 125	Endometrioid carcinoma Grade 1
	8	968	323	Del A	Stop 343	

11	5	349–351	117	Ins A	Stop 125	Endometrioid carcinoma Grade 1
	8	968	323	Del A	Stop 343	

12	5	462	154	TTC to TTT	No change	Endometrioid carcinoma Grade 1
	8	843	281	CCA to CCG	No change	

13	5	349–351	117	Ins A	Stop 125	Endometrioid carcinoma Grade 1

14	2	158	53	Ins C	Stop 62	Endometrioid carcinoma Grade 1
	5	362	121	GCA to GTA	Ala to Val	
		462	154	TTC to TTT	No change	
	7	777	259	CAC to CAT	No change	
	8	968	323	Del A	Stop 343	

15	6	518	173	CGC to CAC	Arg to His	Endometrioid carcinoma Grade 1
	8	968	323	Del A	Stop 343	

16	5	388	130	CGA to GGA	Arg to Gly	Endometrioid carcinoma Grade 1

17	3	204	68	TAC to TAG	Stop 68	Endometrioid carcinoma Grade 1

18	5	389	130	CGA to CTA	Arg to Leu	Endometrioid carcinoma Grade 1

19	5	389	130	CGA to CTA	Arg to Leu	Endometrioid carcinoma Grade 1
	7	691–693	231	Del C	Stop 255	
	8	968	323	Del A	Stop 343	

20	5	389	130	CGA to CTA	Arg to Leu	Endometrioid carcinoma Grade 1

21	5	318	106	GAA to GAG	No change	Endometrioid carcinoma Grade 3

22	1	14	5	ATC to AGC	Ile to Ser	Endometrioid carcinoma Grade 3
	6	511	171	CAG to TAG	Stop 171	
	8	968	323	Del A	Stop 343	

23	5	370	124	TGT to GGT	Cys to Gly	Endometrioid carcinoma Grade 3

24	5	389	130	CGA to CCA	Arg to Pro	Endometrioid carcinoma Grade 3

**Table 3:** The most frequent mutations in endometrial samples.

	Frequency			
	Hyperplasia	CaE type I	CaE type II	Total
A deletion in codon 323 with stop effect in codon 343	3/8 (37.5%)	8/31 (25.8%)	0/0 (0%)	11/39 (27.5%)
Substitution in codon 130	1/8 (12.5%)	6/31 (19.4%)	0/0 (0%)	7/39 (17.9%)
A insertion in codon 117 with stop effect in codon 125	1/8 (12.5%)	3/31 (9.7%)	0/0 (0%)	4/39 (10.3%)

**Table 4:** Frequency of *PTEN* mutations in Slovak, Polish, and Japanese population.

Populations	*PTEN* mutations frequency					
	Hyperplasia	*P* value	Carcinoma	*P* value	Total	*P* value
Slovak	5/15 (33.0%)		19/25 (76.0%)		24/45 (53.3%)	
Polish	2/6 (33.3%)	1.000	27/59 (45.8%)	0.016^*∗∗*^	29/65 (44.6%)	0.439
Japanese	7/73 (9.6%)	0.028^*∗∗*^	13/57 (22.8%)	0.000027^*∗∗*^	29/103 (28.2%)	0.005^*∗∗*^

^*∗∗*^ *P* values < 0.05 were considered statistically significant.

## References

[1] Jemal A., Siegel R., Xu J., Ward E. (2010). Cancer statistics, 2010. *CA Cancer Journal for Clinicians*.

[2] Diba C. S., Pleško I., Hlava P. (2012). *Incidence of Malignant Cancer in Slovak Republic 2007*.

[3] Bokhman J. V. (1983). Two pathogenetic types of endometrial carcinoma. *Gynecologic Oncology*.

[4] Mutter G. L., Lin M.-C., Fitzgerald J. T. (2000). Altered PTEN expression as a diagnostic marker for the earliest endometrial precancers. *Journal of the National Cancer Institute*.

[5] Li J., Yen C., Liaw D. (1997). *PTEN*, a putative protein tyrosine phosphatase gene mutated in human brain, breast, and prostate cancer. *Science*.

[6] Steck P. A., Pershouse M. A., Jasser S. A. (1997). Identification of a candidate tumour suppressor gene, MMAC1, at chromosome 10q23.3 that is mutated in multiple advanced cancers. *Nature Genetics*.

[7] Lee J.-O., Yang H., Georgescu M.-M. (1999). Crystal structure of the PTEN tumor suppressor: implications for its phosphoinositide phosphatase activity and membrane association. *Cell*.

[8] Tamura M., Gu J., Takino T., Yamada K. M. (1999). Tumor suppressor PTEN inhibition of cell invasion, migration, and growth: differential involvement of focal adhesion kinase and p130(Cas). *Cancer Research*.

[9] Di Cristofano A., Pandolfi P. P. (2000). The multiple roles of PTEN in tumor suppression. *Cell*.

[10] Govatati S., Kodati V. L., Deenadayal M., Chakravarty B., Shivaji S., Bhanoori M. (2014). Mutations in the PTEN tumor gene and risk of endometriosis: a case-control study. *Human Reproduction*.

[11] Silverberg S. G., Kurman R. J., Nogales F., Mutter G. L., Kubik-Huch R. A., Tavassoli F. A., Tavassoli F. A., Devilee P. (2003). Epithelial tumors and related lesions. *WHO Classification of Tumours, Pathology and Genetics, Tumours of the Breast and Female Genital Organs. Tumours of the Uterine Corpus*.

[12] Risinger J. I., Hayes A. K., Berchuck A., Barrett J. C. (1997). PTEN/MMAC1 mutations in endometrial cancers. *Cancer Research*.

[13] Risinger J. I., Hayes K., Maxwell G. L. (1998). PTEN mutation in endometrial cancers is associated with favorable clinical and pathologic characteristics. *Clinical Cancer Research*.

[14] Simpkins S. B., Peiffer-Schneider S., Mutch D. G., Gersell D., Goodfellow P. J. (1998). PTEN mutations in endometrial cancers with 10q LOH: additional evidence for the involvement of multiple tumor suppressors. *Gynecologic Oncology*.

[15] Maxwell G. L., Risinger J. I., Gumbs C. (1998). Mutation of the PTEN tumor suppressor gene in endometrial hyperplasias. *Cancer Research*.

[16] Lin W. M., Forgacs E., Warshal D. P. (1998). Loss of heterozygosity and mutational analysis of the PTEN/MMAC1 gene in synchronous endometrial and ovarian carcinomas. *Clinical Cancer Research*.

[17] Kong D., Suzuki A., Zou T. T. (1997). PTEN1 is frequently mutated in primary endometrial carcinomas. *Nature Genetics*.

[18] Tashiro H., Blazes M. S., Wu R. (1997). Mutations in PTEN are frequent in endometrial carcinoma but rare in other common gynecological malignancies. *Cancer Research*.

[19] Konopka B., Janiec-Jankowska A., Czapczak D. (2007). Molecular genetic defects in endometrial carcinomas: microsatellite instability, PTEN and beta-catenin (CTNNB1) genes mutations. *Journal of Cancer Research and Clinical Oncology*.

[20] Kurose K., Bando K., Fukino K., Sugisaki Y., Araki T., Emi M. (1998). Somatic mutations of the *PTEN/MMAC1* gene in fifteen Japanese endometrial cancers: evidence for inactivation of both alleles. *Japanese Journal of Cancer Research*.

[21] Sun H., Enomoto T., Fujita M. (2001). Mutational analysis of the PTEN gene in endometrial carcinoma and hyperplasia. *The American Journal of Clinical Pathology*.

[22] Konopka B., Janiec-Jankowska A., Paszko Z., Goluda M. (2004). The coexistence of ERBB2, INT2, and CMYC oncogene amplifications and PTEN gene mutations in endometrial carcinoma. *Journal of Cancer Research and Clinical Oncology*.

[23] Konopka B., Paszko Z., Janiec-Jankowska A., Goluda M. (2002). Assessment of the quality and frequency of mutations occurrence in PTEN gene in endometrial carcinomas and hyperplasias. *Cancer Letters*.

[24] http://stats.oecd.org/index.aspx?DataSetCode=HEALTH_STAT#.

[25] http://faostat3.fao.org/compare/E.

[26] Arem H., Gunter M. J., Cross A. J., Hollenbeck A. R., Sinha R. (2013). A prospective investigation of fish, meat and cooking-related carcinogens with endometrial cancer incidence. *British Journal of Cancer*.

